# Antiretroviral treatment failure and associated factors among HIV patients on first-line antiretroviral treatment in Sekota, northeast Ethiopia

**DOI:** 10.1186/s12981-020-00294-z

**Published:** 2020-07-10

**Authors:** Jemberu Nega, Solomon Taye, Yihenew Million, Chaturaka Rodrigo, Setegn Eshetie

**Affiliations:** 1 Department of Medical Microbiology, School of Biomedical and Laboratory Sciences, College of Medicine and Health Sciences, University of Gondar, PO Box 196, Gondar, Ethiopia; 2 Department of Pathology, Faculty of Medicine, University of New South Wales, Sydney, New South Wales Australia

**Keywords:** Antiretroviral treatment failure, Risk factors, Northeast Ethiopia

## Abstract

**Background:**

Antiretroviral treatment has played a pivotal role in the reduction of HIV/AIDS-related morbidity and mortality. However, treatment options can be impaired by the development of antiretroviral treatment failure. Regular monitoring of the Human Immunodeficiency Virus treatment outcome via viral load tests is the key approach. There is a scarcity of information about HIV treatment failure and risk factors in the study area. Therefore, the study was aimed to assess antiretroviral treatment failure and associated factors among patients on first-line antiretroviral treatment at Tefera Hailu Memorial Hospital, Sekota, northeast Ethiopia.

**Methods:**

A hospital-based cross-sectional study was conducted on 295 patients on first-line antiretroviral treatment from Nov. 2018 to Apr. 2019. Socio-demographic and clinical variables were collected using a pretested questionnaire, and blood specimen was collected for PCR viral load and CD4 + cell count estimation. Data were entered into Epi-Info and exported to SPSS for analysis. A binary logistic regression model was used to identify associated factors, and *P* value < 0.05 was considered as statistically significant.

**Results:**

Of the 295 subjects on first-line ART, 49 (16.6%) and 18 (6.1%) experienced virological and immunological failures, respectively. The failure of the former was associated with poor adherence (AOR: 6.367, P < 0.001), CD4 + count < 500 cells/µL (AOR: 4.78, P = 0.031) and shorter (6–24 months) duration on ART (AOR: 0.48, P = 0.048), while poor treatment adherence (AOR: 11.51, P = 0.012) and drug interruption (AOR: 6.374, P = 0.039) were the independent risk factors for latter. Immunological tests to predict virological failures showed as sensitivity, specificity, PPV, and NPV were 20.4%, 96.7%, 55.5%, and 86.0%, respectively.

**Conclusions:**

The rate of ART failure was considerably high. Poor adherence, low CD4 + count, prolonged ART, and drug interruption were found to be the most predictor variables for virological and immunological failures. The discrimination power of the immunological parameter was low in comparison to virological measurements as standard methods. Therefore, the study highlighted the need for more attention and efforts to curb associated factors and maximize virological tests for monitoring treatment failures.

## Background

Following the epidemic of HIV/AIDS, the global expansion of the combination antiretroviral treatment (ART) has been the primary contributor to the 51% decline AIDS-related deaths, from a peak of 1.9 million (1.4–2.7 million) in 2004 to 0.94 million (0.67–1.3 million) in 2017. The scaling up of ART was a critical turning point in the clinical management of the HIV/AIDS disease and the gradual evolution of HIV infection into a chronic non-fatal condition by restoring the immune function and suppressing the virus to an undetectable level. This resulted in preventing the transmission of the virus to other uninfected individuals.

Globally, 21.7 million (59%) people living with HIV/AIDS (PLHA) accessed treatment in 2017 and Africa accounted for 15.3 million on ART [1]. In Ethiopia, ART began in 2003 and free ART was launched in 2005. By the test and treat all patients policy of WHO 2016, above 73,800 HIV positive patients required ART in Ethiopia. However, only near half, 426,000, took ARV nationwide [2–4]. Despite the WHO recommended viral load monitoring to ensure viral load suppression and early detect ART treatment failure, large gaps remain in global access, particularly in low- and middle-income countries and rural areas. Individual-level viral load is the recommended measure of antiretroviral therapy efficacy; it indicates treatment adherence and the risk of transmitting HIV [5].

Ethiopia has adopted the WHO plan “End the AIDS epidemic by 2020 to achieve the target of the three 90%’s (know HIV status-get treatment-viral suppression)” ambitious plan. However, due to inaccessibility, costs and technical demands of the HIV RNA test, immunologic (CD4 + T cell test) is still widely recommended in Ethiopia for monitoring treatment failures. But immunologic parameters have a lower accuracy of identifying virologic failures, leading to premature changes or continuous uses of failed regimens. This leads to more complex HIV drug failures, and the health impact increases morbidity and mortality rates in settings where virologic tests are not available.

There has been a marked paucity of information on ART failure and associated risk factors in Ethiopia, including in the study area. Besides, the lack of early detection of the virological failure is often coupled with delays in switching to efficacy drugs. Such conditions weakened the ability to prevent treatment failure and led to accumulation of further HIV-DR at individual and population level. Therefore, this study was conducted to assess the extent of antiretroviral treatment failure and its associated factors among HIV patients on first-line ART at Tefera Hailu Memorial Hospital, Sekota, northeast Ethiopia.

## Methods

### Study area

The study was conducted at Tefera Hailu Memorial Hospital in Sekota town, the capital of Waghimra zone, east Amhara region, 720 km north of Addis Ababa and 320 km north of Dessie. The hospital provided ART and referral case services to nearly half amillion people. The hospital reported that about 1760 patients started ART until October 2018, and that 889 were on treatment at that moment. The hospital laboratory had a separate section for CD4 + count and an estimated 2000 blood specimens were delivered for the purpose annually from the hospital and the surrounding health centers. Blood specimens were collected for viral load determination, processed and made ready for transport to Dessie branch of the Amhara Public Health Institute.

### Study design and population

A hospital-based cross-sectional study was conducted on 295 adolescent and adult PLHA patients who were on first line ART follow ups at Tefera Hailu Memorial Hospital from Nov. 2018 to Apr. 2019. Systematic random sampling technique was used to enroll the participants Sample size was determined using the single population proportion formula, with the following assumption, n = sample size, d = 0.05; P = 0.222, which was reported in the previous study in Gondar [6] and Z α/2 = 1.96 by assuming a 95% confidence level. The minimum sample size was (n = 266), and by considering a 10% non-response rate the final sample is 295.

### Inclusion criteria

All adolescent and adult patients who were on first-line ART and had at least a six-month follow up and volunteerd to participate were included in the study, while, patients who were on second-line ART and had incomplete data before the study were excluded.

### Study variables

Antiretroviral treatment failure defined according to the WHO 2016 guideline was the outcome variable [2]. Age, gender, educational level, occupation, marital status, distance from home to clinic, alcohol usage, disclosure, and monthly income were socio-demographic variables. WHO clinical stages, CD4 + counts and ART regimen (baseline, current status), level of adherence, drug interruption, functional status, ART dose, frequency of drug substitute, tuberculosis in the course of therapy, duration of ART, and HIV prophylaxis were clinical variables.

### Definition of terms

Virological failure is defined as when antiretroviral therapy (ART) fails to suppress viral replication to lower than 1000 copies/mL, while immunological failure is a fall of CD4 + count below 250 cells/µL following clinical failure, or persistent CD4 + count below 100 cells/μL [7].

### Socio-demographic and clinical data collection

Socio-demographic and clinical (baseline, follow up and current patient status) data were collected by expert nurses from patient medical files and face to face interviews using a structured pre-tested questionnaire. The questionnaire was adapted from the WHO risk assessment tool for HIV treatment failure and was developed by using literature, on risk assessment for HIV treatment failure.

### Specimen collection and processing

Expert laboratory staff members collected 4 mL and 2 ml of whole blood into plasma preparation tube (PPT) and ethylenediaminetetraacetic acid (K3 EDTA) tube, for viral load test and CD4 counts, respectively, from each participants. After 30 min, PPT was centrifuged at 1500 rpm for 10 min to separate plasma (top), in between separator gel and the cell (bottom). Then, the plasma containing PPT was transported in a triple package sample transportation box at 2–8 ℃ to Amhara Hublic Health Institute Dessie center, by an orientated laboratory assistant.

### Determination of CD4 + T lymphocyte

The CD4 + T cell count determination was carried out using 50 µl of EDTA whole blood added to the cartridge and run by the Becton–Dickinson Biosciences (BD) Fluorescent Activated Cell Sorter (FACS) Presto near patient machines. In the analysis, the software identifies the T-Lymphocyte populations and calculates the absolute counts of CD4 + cells and results printed out.

### Determination of viral load

RNA extraction from 0.2 mL of plasma and amplification reagent (master mix) preparation was done by using the Abbott m2000sp HIV-1 assay. Then both extracted RNA and master mix were dispensed into a 96 deep well plate (made ready for amplification). In the Abbott m2000rt analyzer extracted RNAs were changed to complementary deoxyribonucleic acid (DNA) by reverse transcriptase polymerase chain reaction (RT-PCR), and DNA was continued for amplification. Then, the DNA product was measured using the Abbott m2000rt quantitative Real-Time HIV-1 assay with HIV-1 RNA detection level depend on the amount of sample ranging 40 to 10 × 10^6^ copies/ml.

### Quality control

The quality of the data was confirmed by pre-testing the questionnaire on 5% of respondents other than those involved in the actual study and by proper designing. Before beginning the actual data collection, training was given to data collectors, and the questionnaire was reviewed and checked. Data collectors were supervised during the process and given feedback by the principal investigator every morning. The whole process of work was assured and approved by advisors.

The Abbott Real-time HIV-1 (m2000sp) assay internal control an RNA sequence unrelated to the HIV-1 target sequence was introduced into each specimen at the beginning of sample preparation. This unrelated RNA sequence was simultaneously amplified by RT-PCR and served as an internal control (IC) to demonstrate that the process proceeded correctly for each sample. Also, negative, low positive and high positive controls were used. For the CD4 test, the FACS Presto machine had internal performance control. Both viral load and CD4 tests were done by experts who used standard operating procedures.

### Data analysis and interpretation

Data were cleaned and checked for completeness and entered into EPI-Info version 7 and exported to SPSS version 20 for analysis. Descriptive statistics (frequencies, percentages, medians, and ranges) were used to describe results. The binary logistic regression analysis was used to determine univariable associations with ART treatment failures. All factors (P ≤  0.2) were finally tested by the multivariable logistic regression for adjusted odds ratios to decrease confounding errors of the independent variables for treatment failure. P-value < 0.05 was considered as statistically significant for treatment failure. Using a two-by-two contingency table, sensitivity, specificity, positive predictive value (PPV), and negative predictive value (NPV), were calculated to determine the validity of the immunological tests for predicting virological failures. Data were also prepared by using tables. The virological and immunological treatment failures were defined according to the WHO 2016 guideline for the use of ART drugs.

## Results

### Sociodemographic information

A total of 295 HIV positive individuals, 169 (57%) of whom were female, on first-line ART regimens participated. The median age of the participants was 37 years (IQR: 30–43), and the majority, 115 (39%), were aged between 30 and 39; 129 (43.7%) were married; nearly three-fourths, 215 (72.9%), attended primary school and above; 143 (48.5%) were self-employed; distance from home to hospital was > 10 km; 79 (27%) of the participants were rural dwellers (Table 1).

**Table 1:** The distribution of virological failure per socio-demographic factors at Tefera Hailu Memorial Hospital, Sekota, northeast Ethiopia, 2019

Variables	Category	N = 295	% From N	VTF = 49	% From failure
Age	10–19	20	6.8	8	16.33
	20–29	42	14.2	7	14.29
	30–39	115	39	17	34.69
	40–49	89	30.2	14	28.57
	50 +	29	9.8	3	6.12
Gender	Female	169	57	28	57.14
	Male	126	43	21	42.86
Marital status	Single	54	18.3	15	30.61
	Married	129	43.7	20	40.82
	Divorced	76	25.8	10	20.41
	Widowed	36	12.2	4	8.16
Education status	Not read/write	80	27.1	9	18.37
	Primary	108	36.6	24	48.98
	Secondary	67	22.7	13	26.53
	Above secondary	40	13.6	3	6.12
Occupational status	Gov. employee	75	25.4	8	16.33
	Self-employee	143	48.5	24	48.98
	Unemployed	43	14.6	12	24.49
	Farmer	34	11.5	5	10.20
Alcohol use	No	246	83.4	42	85.71
	Yes	49	16.6	7	14.29
Distance to ART clinic	< 10 km	216	73.2	36	73.47
	> 10 km	79	26.8	13	26.53

^*VTF* virological treatment failure, *N* total participants, *ETB* Ethiopia Birr, alcohol never allowed for patients on ART, age category according to Ethiopia ART guideline 2017^

### Clinical information

The baseline and current median CD4 + counts were 198 (IQR: 135–314) and 414 cells/µL (IQR: 309–544), respectively. Participants were on first-line ART with a median follow up time of 83 months (IQR 53–116). At ART initiation, 183 (62.37%) patients were ambulatory by their functional status, and 112 (38%) were baseline clinical stage III and IV. In contrast, during data collection, only 2.03% of the participants were in clinical stage III and IV. Presentations were not even observed among the respondents (Table 2).

**Table 2:** The distribution of virological failure per clinical factors at Tefera Hailu Memorial Hospital, Sekota, northeast Ethiopia, 2019

Variables	Category	N = 295	% from N	VTF = 49	%
Current ART regimen	1c	53	18.0	16	32.65
	1d	82	27.8	12	24.49
	1e	135	45.8	15	30.61
	1f	16	5.4	2	4.08
	4c	9	3.1	4	8.16
Baseline ART regimen	1a	26	8.8	4	8.16
	1b	40	13.6	8	16.33
	1c	37	12.5	8	16.33
	1d	68	23.1	12	24.49
	1e	110	37.3	11	22.45
	4c	14	4.7	6	12.24
Drug substitution	None	208	70.5	31	63.27
	Once	87	29.5	18	36.73
Current CD4 result	≤ 100	23	7.8	14	28.57
	101–250	86	29.2	20	40.82
	251–500	88	29.8	11	22.45
	501 +	98	33.2	4	8.16
Current clinical stage	I	265	89.8	34	69.39
	II	24	8.13	12	24.49
	III	6	2.03	3	6.12
Baseline clinical stage	I	59	20.0	7	14.29
	II	124	42.0	21	42.86
	III	98	33.2	18	36.73
	IV	14	4.7	3	6.12
The dose of ART per day	Once	131	44.4	15	30.61
	Twice	164	55.6	34	69.39
Drug interruption	No	261	88.5	37	75.51
	Yes	34	11.5	12	24.49
Drug adherence	≥ 95%	189	64.1	11	22.45
	< 95%	106	35.9	38	77.55
Prophylaxis use	No	285	96.6	44	89.80
	Yes	10	3.4	5	10.80
Baseline function status	Bedridden	28	9.5	7	14.29
	Ambulatory	184	62.4	28	57.14
	Working	83	28.1	14	28.57
TB history	No	242	82	36	73.47
	Yes	53	18	13	26.53
Disclosure	No	71	24.1	10	20.41
	Yes	224	75.9	39	79.59
Duration/month on ART	6–24	29	9.8	1	2.04
	25–48	39	13.2	4	8.16
	49–72	57	19.3	7	14.29
	73 +	170	57.6	37	75.51
Baseline CD4 result	≤ 100	150	50.8	26	53.06
	101–250	88	29.8	15	30.61
	251–500	28	9.5	3	6.12
	501 +	29	9.8	5	10.20

^1a = d4T/3TC/NVP, 1b = d4T/3TC/NVP, 1c = AZT/3TC/NVP, 1d = AZT/3TC/EFV, 1f = TDF/3TC/NVP, 4c = AZT/3TC/NVP, *VTF* virological treatment failure, *N* total participants, *TB* tuberculosis^

At the time of treatment initiation, different classes of first-line ART drugs were prescribed; patients on azidothymidine (AZT), tenofovir disoproxil fumarate (TDF), and stavudine (d4T) based regimens were 119 (40.3%), 110 (37.3%) and 66 (22.4%), respectively. As illustrated in (Table 2), during the implementation of the study, different types of first-line ART regimens, like AZT-based 144 (48.8%) regimens were given to patients. On the other hand, TDF-based regimens were prescribed to 151 (51.2%) patients; 87 (29.5%) of the participants have received substitution (other first lines) for different reasons; for instance, d4T was outdated due to physiological toxicity.

### Virological treatment failure and related risk factors

The prevalence of virological failure among respondents was 49 (16.6%; 95% CI 12.5–20.9), of whom 28 (57.1%) were females. On the other hand, effective viral suppression, < 1000 copies/ml, was noted among 246 (83.4%) of the participants.

The multivariable logistic regression showed that poor drug adherence (< 95%) was identified as one of the independent risk factors for virological treatment failure. Patients with poor adherence experienced just above six times higher odds to develop treatment failure at (AOR = 6.367, 95% CI 2.35–17.2, P < 001). participants with CD4 + counts < 500 cells/µL were almost five times more likely to develop virological treatment failure (AOR: 4.78, 95% CI 1.15–19.77), P = 0.031) in comparison with those who had more CD4 + count. Furthermore, treatment duration was also another predictor variable; only patients who followed treatments for 6–24 months had the chance to minimize treatment failure by nearly 50% (AOR: 0.48 95% CI 0.012–0.973, P = 0.048) compared to those on treatment for over 72 months (Table 3).

**Table 3:** Associated risk factors with virological failure at the Tefera Hailu Memorial Hospital, Sekota, northeast Ethiopia, 2019

Variables	Category	COR (95% CI)	P-V	AOR (95% CI)	P-V
Age	10–19	5.78 (1.3–25.71)	0.021*	4.39 (0.2–116.5)	0.37
	20–29	1.73 (0.41–7.35)	0.45	2.87 (0.32–25.4	0.34
	30–39	1.50 (0.41–5.52)	0.54	1.12 (0.16–7.72)	0.91
	40–49	1.62 (0.43–6.08)	0.47	2.36 (0.35–15.9)	0.37
	50 +		1		1
Education status	Not read/write	1.56 (0.4–6.13)	0.52	0.97 (0.094–83.03)	0.98
	Primary	3.52 (1.0–12.43)	0.05	1.58 (0.195–12.9)	0.66
	Secondary	2.97 (0.79–11.15)	0.10	2.19 (0.26–18.5)	0.47
	Above secondary		1		1
Current ART regimen	1c	0.54 (0.13–2.28)	0.41	2.1 (0.09–48.9)	0.64
	1d	0.21 (0.05–0.91)	0.037*	0.4 (0.01–12.6)	0.60
	1e	0.16 (0.04–0.64)	0.01*	0.14 (0.003–5.6)	0.29
	1f	0.179 (0.02–1.29)	0.08	0.33 (0.007–17.2)	0.58
	4c		1		1
Baseline ART regimen	1a	0.24 (0.054–1.08)	0.06	0.29 (0.005–15.4)	0.54
	1b	0.33 (0.09–1.24)	0.10	1.13 (0.02–76.0)	0.95
	1c	0.37 (0.09–1.37)	0.136	0.39 (0.01–15.9)	0.62
	1d	0.28 (0.08–0.97)	0.046*	1.15 (0.02–65.5)	0.94
	1e	0.15 (0.04–0.50)	0.002*	1.18 (0.02–80.3)	0.94
	4c		1		1
Current CD4 result	≤ 100	36.55 (9.9–94.77)	< 0.001*	42.36 (7.43–79.6)	0.000*
	101–250	7.12 (2.33–21.8)	0.001*	12.1 (2.76–53.1)	0.001*
	251–500	3.36 (1.03–10.96)	0.045*	4.78 (1.15–19.77)	0.031*
	501 +		1		
Current clinical stage	I		1		1
	II	6.79 (2.82–16.34)	< 0.001*	1.1 (0.09–13.3)	0.94
	III	6.79 (1.32–35.03)	0.022*	3.15 (0.83–12.0)	0.09
Dose of ART per day	Once	0.49 (0.25–0.95)	0.036*	0.48 (0.06–4.1)	0.50
	Twice		1		
Drug interruption	No		1		1
	Yes	3.30 (1.50–7.24)	0.003*	1.19 (0.37–3.86)	0.77
Drug adherence	≥ 95%		1		1
	< 95%	9.04 (4.37–18.7)	< 0.001*	6.36 (20.35–17.2)	0.000*
Prophylaxis	No		1		1
	Yes	3.88 (10.18–12.77)	0.026*	5.42 (0.98–29.9)	0.053
TB history	Yes	1.86 (0.9–1.80)	0.09	1.53 (0.53–4.45)	0.44
	No		1		1
Immunologic failure	No		1		1
	Yes	7.63 (2.84–20.5)	0.000*	0.06 (0.003–1.5)	0.088
Duration on ART (months)	6–24	0.13 (0.02–0.97)	0.047*	0.48 (0.01–0.97)	0.048*
	25–48	0.41 (0.14–1.23)	0.11	0.37 (0.07–1.87)	.23
	49–72	0.50 (0.21–1.20)	0.122	0.24 (0.06–0.97)	0.06
	73 +		1		1

^1a = d4T/3TC/NVP, 1b = d4T/3TC/NVP, 1c = AZT/3TC/NVP, 1d = AZT/3TC/EFV, 1f = TDF/3TC/NVP, 4c = AZT/3TC/NVP^

^*PV* probability value, *COR* crude odds ratio, *P < 0.05, *CI* confidence interval, *AOR* adjusted odds ratio^

### Immunological treatment failure and related risk factors

Of the 295 participants, 18 (6.1%) (95% CI 3.4–9.5) faced immunological failures, of these 11 (61.1%) were male. Among immunological failed participants, 10 (55.5%) of them had also viral failure and 8 (44.4%) encountered only CD4 + failure. Patients with poor drug adherence were intriguingly found to be 11.51 times more likely to experience immunological failure (AOR: 11.51, 95% CI = 1.72–77.3, P = 0.012) compared to those who adhered (> 95%). Similarly, patients who interrupted drugs had 6.374 times higher odds of immunological failure (AOR: 6.374, 95% CI 1.093–37.165, P = 0.039) than those who did not.

### Validity of immunological tests for virological failure prediction

In the present study, immunologic failure showed low predictive values for detecting the virological failures. Using the two-by-two contingency table, screening immunologic failure was compared to the golden standard virological failure, and the sensitivity, specificity, PPV, and NPV were 20.4%, 96.7%, 55.5%, and 86%, respectively (Table 4).

**Table 4:** The predictive power of immunological criteria to detect virological treatment failure at Tefera Hailu Memorial Hospital, Sekota, northeast Ethiopia, 2019

Immunological failure	Virological failure (> 1000 copies/ml)		Total
	Yes	No	
Yes (< 500 cells/µL)	10	8	18
No (> 500 cells/µL)	39	238	277
Total	49	246	295

^Sensitivity = true positive (10)/true positive (10) + false negative (39)*100 = 20.4 %. PPV = true positive (10)/true positive (10) + false positive (8) * 100 = 55.5%. Specificity = true negative (238)/true negative (238) + false positive (8) * 100 = 96.75%. NPV = true negative (238)/true negative (238) + false negative (39)  * 100 = 86 %^

## Discussion

It is clear that the plasma viral load test is now increasingly used to check the success of patients on antiretroviral therapy since several studies have shown that the risk for HIV transmission is very low when the viral load is lower than 1000 copies/ml. Our study revealed that about 83.4% of the respondents’ viral load was below 1000 copies/ml (suppressed), and most of them had chances to decrease treatment failure, minimize transmission to others and the risks for clinical conditions, indicating that a combination of ART was highly effective for patients’ recover and the decline of opportunistic infection. Significant HIV associated morbidity and mortality had been reported in pre-ART era. Following the introduction of HAART, however, the quality of life and disease related outcomes have been improved especialty in high HIV burden countries like Ethiopia [7].

On the other hand, we reported that the prevalence of virologic treatment failure was 16.6% (95% CI 12.5–20.9) among participants. The result was comparable to reports of previous studies in Gondar (14.7%) [8] and Addis Ababa (19.8%) [9]. Likewise, our report was consistent with findings in Uganda (14.5%) [10], India (12.7%) [11], Shenzhen, China (13.4%) [12], Haiti (14.2%) [13]. On the other hand, our result was higher when compared to the WHO 2015–2020 target of 10% virological treatment failure among HIV-1 positive patients with ART follow-ups [7]. Our finding was also higher than what were reported by other Ethiopian studies (5.3 to 11.5%) [14–17]. The reasons for the higher failure might be poor adherence and longer duration on ART. Variations in virological failure might be related to the WHO guideline which varies over time. For instance, in our study virological failure was defined as viral load above 1000 copy/ml based on the 2016 WHO guideline [7]. However, some researches defined virological failure as a viral load > 400 copy/ml in line with previous WHO guideline [18]. If studies were done on records, the results may be over estimated because patients are tested only when they are suspected to develop treatment failure, not on routine basis.

Patients with poor adherence had above six times higher odds to develop treatment failure (AOR = 6.367, 95% CI 2.355–17.2, P = 001) compared to those who adhere. Poor adherence leads to a low level of antiretroviral effect in the body, and this causes insufficient to suppress viral replication, finally resulting in treatment failure. In addition, individuals with CD4 + count < 500 cells/µL were also nearly five times more likely to develop virological treatment failure (AOR: = 4.78, 95% CI 1.155–19.77, P = 0.031) as opposed to those who had greater CD4 + counts. It is evident that a high degree of viral replication is common among patients with a decreased level of CD4 count and subsequently leads to treatment failure.

In the present study, patients with shorter (6–24 months) treatment duration were found to be 52% less likely to develop treatment failure (AOR: 0.48, 95% CI 0.012–0.973, P = 0.048) in comparison to those with > 72 months on treatment. Reasonably, if the duration of ART increases, the emergence of HIV-DR strain is expected as a result of HIV error-prone replication, mutation rate, and viral recombination [19]. Over time, acquired drug resistance (during on ART) might cause treatment failure; so patients with shorter duration (6–24 months) on ART have a less likely incidences of acquiring drug resistant viruses than those with longer (> 73 month) among exposure to ART. Thus a high burden of a drug-resistant virus with an increased viral load is expected among patients with more months than with lesser months on ART [19, 20]. Besides, the possible reason might be that as the duration of ART increases, the likelihood of poor adherence, drug interruption, and drug side effects also increase.

Furthermore, immunological failure was found to be 6.1% (95% CI 3.4–9.5). The finding was in line with those of studies conducted in Ethiopia and reported 3.8–6.8% [16, 21, 22]. However, it was lower than the results of other studies in Ethiopia 15–21% [9, 14, 23], Tanzania19% [24], and Shenzhen China 18.4% [12]. Our work showed a lower immunological failure than some studies in Ethiopia and abroad. The possible reason for the low result might be the low sensitivity of the test. Immunological failure may also be related to the WHO guideline which varies over time. For instance, in our study immunological failure was defined as a fall of CD4 + count below 250 cells/µL following clinical failure or as a persistent CD4 + count below 100 cells/μL based on the 2016 WHO guideline. However, a former research defined immunological failure as a fall of CD4 + count to baseline or below severe immune suppression (CD4 + count < 200 cells/μL) in line with previous WHO guidelines.

In the current study, immunologic failure had low predictive values for detecting the virologic failures. The reasons might be low prevalence of immunological failure and test inaccuracy. Compared to the golden standard virologic failure immunologic failure, sensitivity, specificity, PPV and NPV were 20.4%, 96.7%, 55.5%, and 86% respectively. Several studies also pointed out the low performance of immunological criteria for prediction of HIV/AID treatment failure [25–27]. The low sensitivity of the immunological criteria for identifying virological failure could result in delayed switching to second-line treatment with the accumulation of resistance mutations; the low positive predictive value may incorrectly identify patients as needing second-line treatment, while they are virologically suppressed.

## Limitations

The finding of the study is limited owing to the fact that specific drug resistance was not confirmed using genotypic method for resistance and susceptibility, for lack of resources. Being a institutional-based study, the result may not also be generalized to wider population. Despite its limitation, however, the work, we believe, provides important information which would be useful for the ART treatment programs in the country.

## Conclusion

In the present study, the burden of antiretroviral treatment failure according to WHO virological criteria was more than the 10% WHO target failure. The Immunological test had low predictive values for detecting treatment failure compared to viral load estimation as a standard method. Poor adherence, CD4 + count < 500 cells/μL and prolonged duration on ART were significant for virological failure. Therefore, our finding indicates the need for more focus and effort from the study area hospital and concerned bodies on significant risk factors to maximize the successes of patients’ health by preventing, enhance early detection and monitoring of antiretroviral treatment failure and control further complications.

## Data Availability

The datasets used and/or analyzed during the study are available from the corresponding author on reasonable request.
